# Micronutrient Depletion in Heart Failure: Common, Clinically Relevant and Treatable

**DOI:** 10.3390/ijms20225627

**Published:** 2019-11-11

**Authors:** Natasa Cvetinovic, Goran Loncar, Andjelka M. Isakovic, Stephan von Haehling, Wolfram Doehner, Mitja Lainscak, Jerneja Farkas

**Affiliations:** 1 Department of Cardiology, University Clinical Hospital Center “Dr. Dragisa Misovic—Dedinje”, 11000 Belgrade, Serbia; ncvetinovic@gmail.com; 2 Institute for Cardiovascular Diseases Dedinje, 11000 Belgrade, Serbia; loncar_goran@yahoo.com; 3 School of Medicine, University of Belgrade, 11000 Belgrade, Serbia; andjelka.isakovic@gmail.com; 4 Department of Cardiology and Pneumology, University of Goettingen Medical Center, DE-37075 Goettingen, Germany; stephan.von.haehling@med.uni-goettingen.de; 5 German Center for Cardiovascular Research (DZHK), partner site Goettingen, DE-37099 Goettingen, Germany; 6 Berlin Institute of Health Center for Regenerative Therapies (BCRT) and Department of Cardiology (Virchow Klinikum), German Centre for Cardiovascular Research (DZHK) partner site Berlin, Charité Universitätsmedizin Berlin, DE-13353 Berlin, Germany; wolfram.doehner@charite.de; 7 Division of Cardiology, General Hospital Murska Sobota, SI-9000 Murska Sobota, Slovenia; 8 Faculty of Medicine, University of Ljubljana, SI-1000 Ljubljana, Slovenia; 9 Research Unit, General Hospital Murska Sobota, SI-9000 Murska Sobota, Slovenia; 10 National Institute of Public Health, SI-1000 Ljubljana, Slovenia

**Keywords:** heart failure, micronutrients, iron, vitamins, trace elements

## Abstract

Heart failure (HF) is a chronic condition with many imbalances, including nutritional issues. Next to sarcopenia and cachexia which are clinically evident, micronutrient deficiency is also present in HF. It is involved in HF pathophysiology and has prognostic implications. In general, most widely known micronutrients are depleted in HF, which is associated with symptoms and adverse outcomes. Nutritional intake is important but is not the only factor reducing the micronutrient availability for bodily processes, because absorption, distribution, and patient comorbidity may play a major role. In this context, interventional studies with parenteral micronutrient supplementation provide evidence that normalization of micronutrients is associated with improvement in physical performance and quality of life. Outcome studies are underway and should be reported in the following years.

## 1. Introduction

Heart failure (HF) is a complex disease with many potential underlying causes, which affects the function of other tissues and is often followed by comorbidities [1], especially in the elderly [2]. Pathogenesis of HF has been elucidated and includes many mechanisms, such as increased hemodynamic overload, ischemic-related dysfunction, ventricular remodeling, excessive neurohormonal stimulation, abnormal cardiomyocyte calcium cycling, excessive or inadequate accumulation of extracellular matrix, accelerated apoptosis, and genetic mutations [1]. Although no single unifying pathogenetic theory can explain HF completely, there is evidence suggesting that declining bioenergy plays a major role [3]. Fatty acids and carbohydrates are the main energy source for cardiomyocytes. The conversion of these macronutrients to biological energy requires micronutrients such as coenzyme Q10, thiamine, riboflavin, etc., which are essential cofactors for energy production, energy transfer, and maintenance of the physiological heart function [4]. Moreover, patients with HF have oxidative stress, inappropriate food intake (Table 1), altered metabolism and intestinal function, and pro-inflammatory status, which leads to a deficiency of micronutrients (e.g., iron, selenium, and zinc) and consequently affects prognosis. This review aims to underline the role of micronutrients in the pathophysiology of HF, their prognostic implications, and the effects of supplementation. We reviewed micronutrients that were most represented in the literature and could potentially have the most beneficial effects when supplemented in HF patients, namely B complex vitamins (B1, B2, B6, B12), vitamin C, trace elements selenium, zinc, and iron, coenzyme Q10, with a special attention on vitamin D due to a long history of its research and supplementation, and the abundance of available data.

## 2. Vitamin D

Although many diseases (e.g., cancer, autoimmune disorders, infertility and pregnancy complications, insulin resistance, and type 2 diabetes mellitus) are associated with vitamin D levels, its metabolism, and vitamin D-related genes [12,13], the association between vitamin D and cardiovascular disease is still controversial. Vitamin D regulates numerous processes involved in the pathogenesis of HF, such as cell proliferation and differentiation, apoptosis, oxidative stress, inflammation, endothelial function, vascular calcification, and activation of the renin-angiotensin system [14]. Observational studies have reported that subjects with lower vitamin D levels are under higher risk of developing HF [15,16]. Vitamin D deficiency (Table 2) is one of the most common types of hypovitaminosis worldwide, with a prevalence of almost 50% among the elderly [17], and is the most common vitamin deficiency in HF [18]. It is more common among patients with HF compared with healthy control individuals, independently of age [19]. While 90–99% of elderly HF patients are affected by vitamin D deficiency [17,20,21], severe deficiency is reported in 17–68% of HF patients, dependent on age, gender, functional status, HF severity, and left ventricle ejection fraction (LVEF) [17,20,22]. Pathophysiology of vitamin D deficiency includes reduced food intake and intestinal absorption of nutrients, and limited exposure to solar ultraviolet light (Table 1). Accordingly, both aging and HF predispose patients to hypovitaminosis, and they may have an additive effect on vitamin D level in elderly patients. In light of the above, it is reasonable to evaluate vitamin D status in HF patients separately according to age.

Vitamin D is associated with functional status, illness severity, and prognosis in HF [36]. Vitamin D level positively correlates with cardiopulmonary stress test performance [22] and six-minute walk test distance [37], and negatively with NYHA class [17,20]. In a study of severe HF patients, vitamin D concentration was significantly lower in hospitalized subjects requiring intravenous inotropic agents or left ventricular assist devices, compared with those treated as outpatients [22]. Vitamin D also correlates with NT-proANP [19], NT-proBNP [17,38] and left ventricle ejection fraction (LVEF) [20], which are predictors of prognosis in HF. Furthermore, vitamin D levels independently predicted all-cause mortality in an HF study with an 18-month follow-up [38] and strongly predicted mortality in a study with a longer follow-up period of 4 years [36]. Additionally, severe vitamin D deficiency was found as an independent predictor of death due to HF in a study of 3299 enrolled subjects with suspect coronary artery disease [17], and lower levels of vitamin D independently predicts mortality in end-stage HF patients [39].

Although it has been strongly confirmed that vitamin D plays an important role in the development and prognosis of HF, evidence about the effect of vitamin D supplementation in HF patients is inconsistent. It has been documented that vitamin D supplementation (800 IU/day) might protect people >60 years from HF [40]. A recent meta-analysis, which included 81 interventional studies with vitamin D supplementation in HF patients, has confirmed a positive effect on cardiovascular risk factors such as high blood pressure, dyslipidemia, and inflammation [41]. Thus, results suggest that the required dose of vitamin D for improvements in risk factors is ≥4000 IU (100 g)/day, which is above the tolerable upper intake level for adults and can be associated with toxic side effects [42,43]. Supplementation with lower doses (≤2000 IU/day) in HF patients significantly increased vitamin D levels but had no benefits on LVEF, functional capacity, or quality of life [44,45]. The EVITA, a randomized clinical trial (RCT) that compared vitamin D 4000 IU/day to a placebo in patients with advanced HF and vitamin D deficiency (vitamin D ≤ 75 nmol/L), found no difference in mortality and hospitalization rate. Furthermore, supplementation was associated with a greater need for mechanical circulatory support implant (especially in patients with initial vitamin D concentrations ≥ 30 nmol/L) and higher incidence of hypercalcemia [46]. Accordingly, vitamin D supplementation in HF can be considered but requires caution, especially in patients with no evidence of significant deficiency.

## 3. Other Fat-Soluble Vitamins

Although experimental data have provided evidence that vitamin A regulates the cardiac renin-angiotensin system, and that vitamin A receptors impact the development of diabetes-induced cardiac remodeling and HF in patients with diabetes mellitus [47], the association between vitamin A and HF has not been confirmed in observational studies. Only one study has evaluated vitamin A status in HF, and it found no significant difference between HF patients and sex-matched healthy controls [48]. Dietary vitamin A intake showed no association with HF mortality in the Japan Collaborative Cohort Study, which enrolled 58,696 subjects [49].

Observational studies have found no significant differences in vitamin E status between HF patients and healthy controls [48,50]. The same result was found in a rat model of HF [51]. However, in the Japan Collaborative Cohort Study, high dietary intake of vitamin E was associated with a reduced risk of HF mortality in women but not in men [49]. Although vitamin E supplementation (400 mg/day) reduces oxidative stress in HF patients [52], it has no impact on symptoms, physical functioning, or mortality [53,54,55,56]. Moreover, there are indications that vitamin E treatment may contribute to HF development [54,55].

It is known that vitamin K status is associated with reduced coronary artery calcification and cardiovascular mortality risk. Vitamin K levels, intake, and supplementation have not been evaluated in HF patients, although epidemiological studies suggest a protective role of vitamin K in cardiovascular diseases [57].

## 4. B Vitamins

Thiamine (vitamin B1) is a cofactor in the metabolism of carbohydrates and amino acids and is also essential for aerobic metabolism and adenosine triphosphate (ATP) production. Thiamine deficiency is associated with many cardiovascular diseases, including HF, chronic vascular inflammation, myocardial infarction, and conduction defects [58]. Furthermore, the deficiency can cause HF by depriving the heart of ATP [59]. On the other hand, HF induces thiamin deficiency by increasing urinary excretion due to therapy with loop diuretics, and poor absorption of thiamine due to cardiac cachexia and splanchnic congestion [60,61]. The addition of spironoloctone to loop diuretics has beneficial effects on thiamine levels in HF patients [62].

The prevalence of thiamine deficiency (Table 2) in HF patients is significantly higher compared with non-HF subjects and ranges from 3% in ambulatory settings to 91% in hospital settings [63]. The first observational study that evaluated the effects of thiamine supplementation in HF enrolled only six patients who were treated by intravenous thiamine (100 mg/day) for 7 days [64]. The results confirmed significant improvement in thiamine levels as assessed by LVEF and NYHA, which encouraged further research, but findings were controversial. An RCT using seven days of intravenous thiamine versus a placebo in a double-blind manner, followed by six weeks of oral thiamine (200 mg/day) in all patients, indicated significant improvement of LVEF in the thiamine group after seven days (LVEF increase was 4%), and impressive improvement of 22% in all patients at the end of treatment [65]. An RCT with oral thiamine (300 mg/day), had a small sample size (18 patients) and a short follow-up period (28 days), but confirmed LVEF improvement after supplementation [66]. However, these results were not confirmed in an RCT that enrolled 52 patients. The main difference between these two RCTs with similar designs was the use of diuretic therapy, which significantly influenced thiamine level. In the trial with no LVEF improvement, the proportion of patients who were on furosemide was low (<20%) and spironolactone was prescribed to most patients (>80%) in the thiamine group [62]. Oral supplementation may be less effective than intravenous, due to the impaired enteral absorption in HF. Limitations of all performed studies, and potential cause of inconclusive results, included small sample sizes, short follow-up periods, and subjective measures used as endpoints. Accordingly, further research is needed to elucidate the effects of thiamine supplementation on mortality, hospitalization rate, functional status, and quality of life in HF.

Riboflavin (vitamin B2) is an essential cofactor in cellular energy production, and its deficiency may contribute to the depletion of energy reserves observed in the failing heart. It is water-soluble, has limited tissue storage, and depends on intake (Table 1) and renal excretion. Prevalence of riboflavin deficiency (Table 2) is significantly higher (27%) in HF patients than in healthy controls (2.2%) [67,68]. A study on animal models of HF suggests that riboflavin supplementation significantly improves left ventricular systolic and diastolic function [69]. Further research on HF and riboflavin deficiency in humans is required.

Pyridoxine (vitamin B6) plays an important role in intermediary metabolism, as a cofactor mainly in the metabolism of amino acids, but also of carbohydrates and lipids, as well as in the biosynthesis of neurotransmitters. Pyridoxine deficiency (Table 2) prevalence is also higher in HF patients (38%) than in healthy controls [68]. There are no investigations of isolated pyridoxine supplementation in HF patients.

Cobalamin (vitamin B12) deficiency has been evaluated in the setting of HF, but results are controversial. The largest study evaluating vitamin B12 in HF, with almost 1000 patients, provided unexpected results: vitamin B12 had a weak negative correlation with LVEF and a significant positive correlation with NYHA class [70]. A possible reason for these results is the unclear selection of HF patients: a large number of patients had no systolic dysfunction (mean LVEF were 58% and 65% for men and women, respectively) and no clinical manifestations of HF (about 35% patients were NYHA class I). An observational study found that vitamin B12 deficiency (serum level <200 pg/mL) is relatively rare in HF (5%), and that vitamin B12 level is not related to prognosis [71]. However, the study was not limited to patients with reduced LVEF, and 13% of enrolled patients had LVEF >45%. Although parenteral replacement therapy should be started soon after the vitamin B12 deficiency has been established [72], an interventional study with vitamin B12 supplementation, in addition to folate and vitamin B6, in elderly HF patients (mean age 81 years) suggested no association with NT-proBNP levels [73]. In the future, vitamin B12 should be evaluated in the setting of HF with reduced ejection fraction.

## 5. Vitamin C

Three observational studies [48,50,74] showed significantly lower vitamin C levels among HF patients (mean 39.7 μmol/L, 61 μmol/L, 22 μmol/L, respectively) than in healthy controls, although in all of them, concentrations were above the cutoff for deficiency (Table 2) [75]. In a more recent study, patients with HF had lower vitamin C levels (43.3 μmol/L in NYHA II and 46.8 μmol/L in NYHA III/IV) than the control group had (57.2 μmol/L), but the difference was not significant [67]. Two studies, which determined vitamin C deficiency by a 3-day food diary, found a similar prevalence of the dietary deficiency (39% and 40%, respectively) among HF patients [76,77]. Furthermore, dietary deficiency predicted shorter cardiac event-free survival in both of them. Although some interventional studies have shown that vitamin C supplementation enhances the contractile response to dobutamine [78] and improves endothelial function [79] in HF, there have been no RCTs evaluating vitamin C supplementation in HF. For further research, we propose RCTs that evaluate the effects of vitamin C supplementation on biomarker status, echocardiography parameters, functional status, quality of life, and long-term outcomes in HF patients with confirmed vitamin C deficiency.

## 6. Coenzyme Q10

Coenzyme Q10 (CoQ10) is essential for ATP synthesis, as well as a powerful lipid-soluble antioxidant [80]. Myocardial CoQ10 deficiency has been observed in patients with HF, and it correlates with the severity of symptoms and the LVEF [81]. CoQ10 supplementation also significantly increases its concentration in the blood [82]. Lower levels of CoQ10 (Table 2) are observed in elderly patients and those with more advanced HF [83]. Since 1976, when the first interventional study with CoQ10 administration in HF suggested its therapeutic potential [84], many RCTs have been performed that confirm its benefit in the treatment of HF [85]. CoQ10 significantly improves cardiac output, cardiac index, stroke volume, and LVEF [86,87]. The administration of CoQ10 in patients with end-stage HF awaiting cardiac transplantation significantly improved functional status, clinical symptoms, and quality of life, but there were no changes in echo parameters [88]. An RCT performed 25 years ago that enrolled 641 HF patients (NYHA class III and IV) demonstrated that the addition of CoQ10 to conventional therapy (2 mg/kg daily for 1 year) significantly reduces hospitalization due to HF worsening and the incidence of serious complications [89]. The most recent Q-SYMBIO RCT (CoQ10 100 mg 3 times/day for 2 years vs. placebo) confirmed that CoQ10 as an adjunctive treatment of HF is safe, improves symptoms and functional status, and reduces major adverse cardiovascular events, such as death from HF, sudden cardiac death, and hospitalization due to HF worsening [90]. According to all of the above, we suggest that physicians should consider CoQ10 supplementation in advanced HF, especially if the deficiency is confirmed.

## 7. Selenium

Selenium is an essential nutrient and one of the most important antioxidants in the body. It is found within the body in various selenoproteins, such as glutathione peroxidase (GPx), thioredoxin reductase and selenoprotein P [91]. Moreover, selenium has an important role in converting thyroxin into the biologically active triiodothyronine. This may be an additional mechanism by which low concentrations of selenium compromise cardiovascular conditions [92]. The dietary intake of selenium (Table 1) differs throughout the world. In Europe, due to poor selenium content in the soil, the estimated mean intake of selenium (40 μg/day) [93] is significantly lower than the proposed dose for a normal-weighted Caucasian (75 μg/day), which is needed for optimal function of selenoprotein P [94] and cancer protection [33]. Furthermore, it is well known that selenium deficiency may be a cause of reversible HF, a condition known as Keshan Disease [95]. Also, it has been suggested that patients with HF tend to have lower circulating levels of selenium (Table 2) than individuals free from the condition [43]. A case-control-pair longitudinal study with 11,000 enrolled participants found that subjects with low selenium levels have a higher risk for myocardial infarction and cardiovascular mortality [96], but RCTs with selenium supplementation have found no evidence of cardiovascular protection [97].

The metabolism of selenium and CoQ10 are strongly associated with each other, and a deficiency of one can reduce levels or function of the other [98,99,100]. Accordingly, they are often used and evaluated together. An RCT of an elderly population (mean age 78) who were supplemented with 200 μg/day of organic selenium and 200 mg/day of CoQ10 versus a placebo, found that long-term supplementation reduces NT-proBNP levels and cardiovascular mortality in those with mild to moderate impaired cardiac function [101]. Although an RCT with multiple micronutrient supplementation that included selenium, suggested a beneficial effect on LVEF and quality of life in 30 HF patients (LVEF ≤ 35%, HF due to ischemic heart disease) [102], data about its effects on HF when used alone are insufficient. A recent RCT that compared selenium 200 µg/day with a placebo indicated that supplementation for 12 weeks to HF patients has beneficial effects on insulin metabolism and few markers of cardio-metabolic risk [103].

## 8. Zinc

Zinc, which has antioxidant properties [104], is an essential element for humans and is required for enzyme function, multiple signaling pathways and transcription factors, and is also the second most abundant trace metal in humans [105]. Although zinc is widely distributed in human tissue and has numerous functions, it has an important role in controlling cardiac contractility in cardyomyocites [106].

One of the oldest studies on zinc levels in HF patients evaluated zinc status in 20 younger HF patients (34–64 years), and indicated significantly lower levels in subjects with dilated cardiomyopathy than in healthy controls (74.5 μg/dL or 11.4 μmol/L vs. 93.1 μg/dL or 14.2 μmol/L, respectively) [107]. This finding has been supported by studies on patients with LVEF <40% and NYHA class II-IV [48,108,109,110], and finally confirmed by a recent meta-analysis published in 2018 [111]. Moreover, subgroup analysis found that patients with idiopathic dilated cardiomyopathy had lower zinc levels than control subjects, except for patients with ischemic cardiomyopathy. A recent study enrolled 968 hospitalized patients with decompensated HF who were divided into 3 groups based on serum zinc levels, and found the highest cardiac and all-cause mortality in the third tertile (<62 µg/dL). Serum zinc level was a predictor of cardiac and all-cause mortality, independently of age, gender, comorbidities, medications, other micronutrient levels, B-type natriuretic peptide, and LVEF [112].

Although the role of zinc in cardiovascular medicine has been well represented in molecular research and observational studies in the past few years, there have been no interventional studies or RCTs evaluating zinc supplementation in HF. Two previous studies that included zinc in multiple micronutrient supplements have suggested an association with improvement of cardiac function and quality of life [103,113].

According to the above, further research into zinc supplementation in HF is needed. We propose a study population of elderly patients with advanced HF due to non-ischemic cardiomyopathy and confirmed zinc deficiency, who could have the greatest benefit of the supplementation.

## 9. Iron

Iron is an essential microelement, required for transport, storage and usage of oxygen in humans. In HF, iron deficiency (Table 2) is one of the most common comorbidities, affecting 37–61% of patients [114,115,116]. The deficiency, even before the onset of anemia, can be severe among patients with CHF, aggravating symptoms, quality of life, functional status, and clinical outcomes, and is associated with an increased risk of mortality [114,117,118,119,120]. The 2016 European Society of Cardiology (ESC) guidelines for the diagnosis and treatment of acute and chronic HF recommend that all patients with HF should be tested for iron deficiency [121]. Moreover, intravenous iron, ferric carboxymaltose, is specifically recommended to be considered for the treatment of iron deficiency in HF, in order to alleviate symptoms and improve exercise capacity and quality of life [122,123]. Oral iron supplementation has no benefit in the setting of HF [124,125] and is not recommended by ESC guidelines. The impaired enteral absorption and other conditions characterized by immune activation are considered as causes for the ineffectiveness of oral iron administration. Findings of the IRONOUT HF RCT (oral iron therapy vs. placebo) [124] are in contrast to results from RCTs of intravenous iron repletion (FAIR-HF and CONFIRM-HF) [122,123], although patient populations were similar. Moreover, in the IRONOUT HF oral therapy produced improvement in iron stores, though the improvement was minimal and clinically not significant.

Iron deficiency is also a frequent co-morbidity in HF with preserved EF (HFpEF), which associated with reduced exercise capacity and quality of life, and its prevalence increases with increasing severity of diastolic dysfunction [126]. Beneficial effects of intravenous iron therapy in an animal model of HFpEF [127] encourage further research, which should elucidate the effects of iron therapy in HF with preserved LVEF.

## 10. Conclusions and Future Perspectives

Although micronutrient deficiencies are frequent among HF patients, their role in pathogenesis and treatment has not yet been completely elucidated. Besides iron supplementation, which is recommended by ESC, CoQ10 supplementation has the strongest evidence of benefit in HF. The available data suggest consideration of vitamin D supplementation in cases of confirmed deficiency in patients with HF. Some micronutrients (selenium, zinc, vitamin B2, and vitamin B6) showed improvement of various clinical parameters in HF, but were investigated only in a multiple supplementation setting, while the effects of vitamins B1 and B12 were inconsistent due to unclear selection of patients and poorly defined endpoints. The influence of vitamin C supplementation in HF is yet to be determined. Thus, interventional studies with strict selection criteria and clear endpoints for each micronutrient are still needed to gain better insight into their role in HF. Furthermore, the question remains as to whether the deficiency cutoff values drawn from the general population apply to HF patients; this should be addressed in future research. Supplementation trials should take into account reduced food intake and poor intestinal absorption of nutrients in these patients, and should investigate not only dosage with beneficial effects, but also the routes of administration (i.e., oral or intravenous). In addition, the available data on micronutrient supplementation in patients with HFpEF are scarce and should be thoroughly researched in the future.

## Figures and Tables

**Table 1:** Recommended micronutrient dietary allowance for the general population.

Micronutrient	RDA	Reference
Vitamin D	15–20 µg/day	[5,6]
Thiamin (B1)	1.1–1.2 mg/day	[7]
Riboflavin (B2)	0.9–1.3 mg/day	[8]
Pyridoxine (B6)	1.6–2.0 mg/day	[9]
Cobalamin (B12)	2.0–2.4 µg/day	[10]
Vitamin C	75–90 mg/day	[11]
Coenzyme Q10 *	n.a.	
Selenium	55 µg/day	[10]
Zinc	8–11 mg/day	[11]
Iron	8–18 mg/day	[11]

RDA: recommended dietary allowance. n.a.: not assessed. *: no RDA has been established.

**Table 2:** Serum normal concentrations and definition of deficiency of micronutrients.

Micronutrient	Reference Range	Insufficiency	Deficiency	Reference
Vitamin D	>30 ng/mL >75 nmol/L	21–29 ng/mL 52.5–72.5 nmol/L	<20 ng/mL <50 nmol/L	[23]
Thiamin (B1) *	25–75 ng/mL 75–195 nmol/L			[24]
Riboflavin (B2) ^a^			EGRAC ≥ 1.3	[25]
Pyridoxine (B6) ^b^	5–50 ng/mL 23–223 nmol/L	20–30 nmol/L	<20 nmol/L	[26,27,28]
Cobalamin (B12)	180–950 pg/mL 125–701 pmol/L		<200 pg/mL <148 pmol/L	[29,30]
Vitamin C	>50 µmol/L	10–50 µmol/L	<10 µmol/L	[31]
Coenzyme Q10	0.5–1.7 µmol/L			[32]
Selenium	70–150 ng/mL 0.9–1.8 µmol/L			[33]
Zinc	0.7–1.6 µg/mL 11–25 µmol/L			[34]
Iron	45–160 µg/mL 8–23 µmol/L		<45 µg/mL <8 µmol/L	[35]

*: exact range depends on the laboratory; different cutoff values are used. ^a^: expressed as erythrocyte glutathione reductase activation coefficient (EGRAC). ^b^: measured as pyridoxal phosphate.
